# The Dark Tetrad and Male Clients of Female Sex Work

**DOI:** 10.3389/fpsyg.2020.577171

**Published:** 2020-09-17

**Authors:** Adam C. Davis, Tracy Vaillancourt, Steven Arnocky

**Affiliations:** ^1^Faculty of Education, University of Ottawa, Ottawa, ON, Canada; ^2^Department of Psychology, Nipissing University, North Bay, ON, Canada

**Keywords:** prostitution, sex work, short-term mating strategies, personality, Dark Tetrad

## Abstract

Many scholars have investigated the attitudes, beliefs, motives, and behavior of male clients of female sex workers. However, few have examined individual differences in major dimensions of personality expressed by men who purchase prostitution compared to those who do not. Although several evolutionary psychologists have studied prostitution and those involved in sex work, to our knowledge, none have explicitly considered the utility of an evolutionary personality perspective in trying to understand why particular men pay for sex. In the current mini-review, following other researchers, prostitution is described principally as a form of short-term mating sought primarily by men. We argue that the socially aversive traits embodying the Dark Tetrad (narcissism, Machiavellianism, psychopathy, and sadism) may characterize certain male clients of female sex workers, particularly those consumers expressing the motives of desiring exciting and novel sex with women who are treated with contempt, perceiving prostitution in a business-like manner with little emotional involvement, and seeking to dominate and control sex workers who are viewed as vulnerable and subservient. The traits of the tetrad may also be more prevalent among men who purchase sex from female sex workers in outdoor (e.g., street prostitution) in comparison to indoor settings (e.g., escort agencies).

## Introduction

Direct prostitution (e.g., street prostitution, escort services, and brothels) has been described as a form of sex work that involves an explicit exchange of material goods, favors, and/or services in return for sexual intimacy or erotic acts with no required commitment (Harcourt and Donovan, 2005). This is contrasted with indirect prostitution, wherein the exchange of money for sex may not be the main source of income (e.g., massage parlor workers), where purveyors do not refer to themselves as prostitutes (e.g., “camgirls,” adult film actors/actresses, and exotic dancers), or when people are forced into sex work out of necessity (e.g., survival sex). However, the terminology describing prostitution is a hotly contested matter among scholars (Benoit et al., 2018). Prostitution has been practiced across a myriad of different cultures since ancient times in sex-specific ways, whereby men have tended to be the primary consumers of sex work services offered by women and men (Dylewski and Prokop, 2019). Estimates of the percentage of men who purchase sex cross-culturally vary widely from 9 to 80% (discussed in Farley et al., 2011); however, several investigators have cautioned that previous estimates are likely inflated due to methodological issues (e.g., sampling bias), and that a more conservative estimate below 20% likely typifies men who have ever paid for sex (Månsson, 2004; Pan et al., 2011; Jewkes et al., 2012; Monto and Milrod, 2014; Ondrášek et al., 2018).

In the last decade, an increasing amount of scholarly work has been devoted to studying male clients of female sex workers (discussed in Milrod and Monto, 2017). Many researchers have studied the role of individual differences in attitudes, beliefs, motives, and behavior among men paying for direct and indirect forms of prostitution in comparison to those who do not pay for sex (Pitts et al., 2004; Monto and McRee, 2005; Farley et al., 2011, 2017; Monto and Milrod, 2014; Milrod and Monto, 2017). But few have directly assessed differences in major dimensions of personality (i.e., constellations of enduring individual differences in emotion, motivation, thoughts, and behavior) among those paying for prostitution (Wilson et al., 1992; Xantidis and McCabe, 2000; Sawyer et al., 2001). Several scholars have approached the topic of prostitution using the framework of evolutionary psychology (Burley and Symanski, 1981; Buss and Schmitt, 2001; Salmon, 2008; Prokop et al., 2018; Dylewski and Prokop, 2019). However, to our knowledge, none have explicitly considered the utility of an evolutionary personality perspective in studying male clients of prostitution, which is the focus of the current mini-review.

## Personality, Mate Selection, and Mate Competition

Evolutionary psychologists have emphasized how personality dimensions influence both mate preferences (i.e., intersexual selection) and the ways in which people compete with rivals for mating opportunities (i.e., intrasexual competition; Jonason et al., 2012, 2015; Buunk et al., 2017; Buss and Schmitt, 2019). Because individual differences in personality impact fitness-relevant outcomes and show a high degree of inter-individual variability, heritability, and developmental stability, it is fruitful to consider their potential adaptive value (Nettle, 2006; Buss, 2009). From the perspective of life history theory, personality traits in human and non-human animals are argued to embody resource investment trade-offs (e.g., time, energy, and material assets) between the different components of fitness across the lifespan (e.g., survival, health, reproduction, and parenting) that are shaped by the social-ecological environment and consequently impact life history outcomes (e.g., age of sexual maturity; Figueredo et al., 2007; Wolf et al., 2007; Buss, 2009; Jonason et al., 2010; Simpson et al., 2017; Davis et al., 2019a,b). This life history framework has been applied to personality traits deemed to be socially desirable across cultures, such as honesty-humility (avoidance of manipulating others; Lee et al., 2013; Davis et al., 2019a), but also those dimensions of personality regarded as socially noxious, such as the interrelated traits that constitute the Dark Tetrad (Jonason et al., 2010; McDonald et al., 2012; Holtzman and Donnellan, 2015; Book et al., 2016; Davis et al., 2019b).

## The Dark Tetrad and Short-Term Mating

The Dark Tetrad is a four-variable model of personality that is characterized by ego-centrism and grandiosity (i.e., narcissism), manipulative and cynical tendencies (i.e., Machiavellianism), callousness and antisociality (i.e., psychopathy), as well as taking enjoyment in the pain and suffering of others (i.e., sadism; Buckels et al., 2013; Book et al., 2016; Meere and Egan, 2017; Paulhus et al., 2018). From a life history perspective, investigators have argued that the Dark Tetrad dimensions appear to comprise an organized system of co-adapted traits that facilitate, to varying degrees, investment in early sexual development, short-term mating strategies, risk-taking, exploitation, and aggressive behavior; a so-called “fast life history strategy” (Lalumière et al., 2001; Gladden et al., 2009; Jonason et al., 2010; McDonald et al., 2012; Davis et al., 2019b). This is contrasted with personality traits, such as honesty-humility, that appear to be linked to a “slower life history strategy” whereby resources are invested in producing fewer offspring later on in development, heightened parental care, risk-aversion, as well as greater physical and psychosocial health (Davis et al., 2019a). However, researchers have cautioned that the application of life history theory to human personality has become fractioned from its theoretical origins in ecology and evolutionary biology (Nettle and Frankenhuis, 2019). The fast–slow continuum of life history has also been criticized by some who argue that these strategies do not operate on a single continuum (Holtzman and Senne, 2014). Scholars have further contended that the evolutionary processes that lead to differences between species in life history (Darwinian evolution) are not the same as those that lead to differences in psychological traits among members within a species (e.g., developmental plasticity; Zietsch and Sidari, 2019). Nonetheless, academics maintain that a life history framework is useful for understanding variability in personality traits in human and non-human animals (Wolf et al., 2007; Vonk et al., 2017; Davis et al., 2019b; Young et al., 2019; Del Giudice, 2020).

In previous research, the traits of the tetrad have been associated with, to varying degrees, an earlier sexual debut (Harris et al., 2007), a higher sex drive, impersonal sexual fantasies with multiple partners (Baughman et al., 2014), an unrestricted sociosexual orientation (i.e., a willingness and desire to have sex in the absence of love and commitment; Foster et al., 2006; Holtzman and Strube, 2013; Jones and de Roos, 2017; Tsoukas and March, 2018; Fernández del Río et al., 2019), a higher likelihood of committing infidelity (Jones and Weiser, 2014), and sexual risk-taking (Dubas et al., 2017) that collectively signal heightened investment in short-term mating. Individuals expressing higher levels of the Dark Tetrad personality dimensions are also more likely to engage in aggression (Paulhus et al., 2018), sexually coercion (Koscielska et al., 2019), and criminal behavior (e.g., vandalism, illegal substance use, and assault; Edwards et al., 2017; Pfattheicher et al., 2019). Deficits in affective empathy (i.e., a diminished capacity to feel the emotions of another) have also been linked to higher levels the Dark Tetrad traits (Jonason and Krause, 2013; Jonason et al., 2013; Sest and March, 2017; Heym et al., 2019). It has been argued that to feel empathy for another, it is necessary to perceive that others have minds like our own (i.e., mentalization). Failing to attribute minds to others can result in not seeing other people as fully human (i.e., dehumanization), which may facilitate interpersonal aggression and violence (Fiske, 2009; Farley et al., 2017). These dynamics may be especially apparent among those with elevated levels of Dark Tetrad traits (Bastian, 2019).

## Prostitution, Short-Term Mating, and the Evolution of Economic Exchange

Evolutionary scholars have argued that prostitution qualifies a form of short-term mating because it tends to involve an explicit exchange of goods (e.g., money, jewelry, and/or drugs) for temporary and impersonal sexual intimacy (Burley and Symanski, 1981; Buss and Schmitt, 2001; Salmon, 2008; Meskó et al., 2014; Prokop et al., 2018; Dylewski and Prokop, 2019). Previous researchers have shown how several non-human animals exchange material resources for sexual opportunities and vice versa. Female purple-throated carib hummingbirds (*Eulampis jugularis*) trade sex for access to feeding sites that are vigilantly guarded by more dominant males (Wolf, 1975). Female Adélie penguins (*Pygoscelis adeliae*) engage in extra-pair copulations with unpaired males in exchange for stones that are required for nest construction (Hunter and Davis, 1998). Female chimpanzees (*Pan troglodytes*) provide sex in return for calorie-rich meat from males that hunt (Gomes and Boesch, 2009). Although money is a human cultural artifact, brown capuchin monkeys (*Cebus apella*) are capable of learning the validity of symbolic currency and may trade tokens for sex (Chen et al., 2006; Santos and Rosati, 2015; De Petrillo et al., 2019). These studies highlight how prostitution is not unique to humans and that monetary transactions can be understood from an evolutionary perspective.

## Individual Differences Among Male Clients of Female Sex Workers

Cross-culturally, men who pay for sex report having more sexual partners than men who have never purchased sex (Pitts et al., 2004; Monto and McRee, 2005; Ward et al., 2005; Schei and Stigum, 2010; Ompad et al., 2013; Monto and Milrod, 2014; Farley et al., 2017). Male clients also tend to report an earlier sexual debut than non-clients (Schei and Stigum, 2010; Ompad et al., 2013; Rich et al., 2018) and display a preference for having sex with a variety of partners, as well less relational and committed sex (Xantidis and McCabe, 2000; Farley et al., 2017). Furthermore, male clients of prostitution express more permissive attitudes toward extramarital sex, they think about sex more frequently, report a higher frequency of masturbating (Monto and McRee, 2005; Monto and Milrod, 2014), and access pornography more often than men who do not pay for sex (Monto and McRee, 2005; Farley et al., 2012). Male clients have further been shown to engage in more sexual risk-taking and have a higher likelihood of acquiring and transmitting sexually transmitted illnesses than non-buyers (Ward et al., 2005; Schei and Stigum, 2010; Pan et al., 2011; Ompad et al., 2013; Rich et al., 2018; Seidu et al., 2019). Compared to men who have never bought sex, male clients also display less empathic accuracy (i.e., accurately inferring the thoughts and feelings of another) toward female sex workers than non-clients (Farley et al., 2011, 2017). Purchasing sex has also been associated with rape myth acceptance among men (Cotton et al., 2002), as well as the perpetration of intimate partner violence (Raj et al., 2008; Decker et al., 2009), sexually coercive behavior (Farley et al., 2011, 2017), and rape against non-prostituting women (Monto and McRee, 2005; Jewkes et al., 2006).

Across samples of arrested offenders and non-offenders from different cultures, a very consistent set of reasons regarding why men purchase sex from women have been identified (McKeganey, 1994; Xantidis and McCabe, 2000; Vanwesenbeeck, 2001; Cotton et al., 2002; Månsson, 2004, 2006; Pitts et al., 2004; Lowman and Atchison, 2006; Monto, 2010; Milrod and Monto, 2012, 2017; Farley et al., 2017; Ondrášek et al., 2018). However, caution should be exercised when trying to extrapolate findings from offenders to non-arrested male clients of sex work because these individual differ in important ways (e.g., in terms of demographic characteristics; Monto and McRee, 2005; Monto and Milrod, 2014), which may affect the degree to which they express certain motives for buying sex. These key general motives include: wanting novel, exciting, and forbidden sex with a variety of female sex workers who are treated with contempt to satisfy their sexual urges; seeking specific sexual acts that dating or romantic partners are unwilling or unlikely to provide; perceiving sex in a business-like manner without emotional involvement that is less complicated than dating and romantic relationships; a desire to dominate and control female sex workers who are perceived as vulnerable and subservient; and seeking comfort, companionship, love, and intimacy. The results described in this section support the idea that a majority of male clients of female sex workers express heightened short-term mating proclivities, but that the motives guiding a subgroup of men purchasing prostitution may actually signal long-term mating effort (e.g., wanting companionship).

Few researchers have directly assessed major dimensions of personality among male clients of female sex workers. In Zimbabwe, men who had previously been clients of prostitution reported higher levels of impulsivity, pleasure seeking, and ego-defensiveness (Wilson et al., 1992). Australian male clients of brothels purchasing services from female sex workers expressed higher levels of sensation-seeking (i.e., eagerness in seeking out novel and stimulating activities) than non-clients (Xantidis and McCabe, 2000). Xantidis and McCabe (2000) also found that male clients espousing motives in line with viewing sex as a business transaction, were significantly higher in sensation-seeking than customers seeking romance and companionship. American men who bought prostitution services reported heightened levels of hostile masculinity, which is argued to be a personality profile embodying hostility and cynicism toward women, and attitudes that justify aggression toward and the domination of women (Farley et al., 2017). Among American men arrested for prostitution, those who endorsed inaccurate beliefs about prostitution (e.g., “prostitutes make a lot of money”), scored higher on cynicism (misanthropy and interpersonal distrust) and antisocial practice (criminal behavior and lawlessness), and lower on self-esteem (Sawyer et al., 2001).

## Personality, Prostitution, and the Dark Tetrad

**FIGURE 1:**
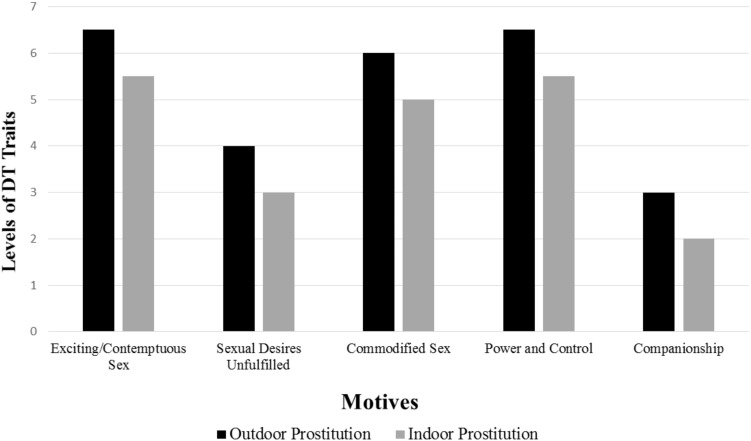
Dark Tetrad, Motives, and Type of Prostitution Service. Hypothetical expression of Dark Tetrad traits along a scale from 1 (*low*) to 7 (*high*) among male clients of female sex works by motive and type of prostitution service.

There are several lines of evidence discussed in the preceding sections that support the argument that the Dark Tetrad personality characteristics may be relevant in understanding why certain men pay for sex. Like many male clients of prostitution, individuals higher on the Dark Tetrad traits tend to express a penchant for short-term and impersonal sex (Holtzman and Strube, 2011, 2013; Jonason et al., 2012, 2015; Tsoukas and March, 2018; Fernández del Río et al., 2019), sexual risk-taking (Dubas et al., 2017), a desire for stimulation and novelty (Baumeister and Campbell, 1999; Carter et al., 2014), impulsivity (Jonason and Tost, 2010; Jones and Paulhus, 2011), greater rape myth acceptance (Jonason et al., 2017), lower emotional empathy (Jonason and Krause, 2013; Jonason et al., 2013; Sest and March, 2017; Heym et al., 2019), as well as the perpetration of interpersonal aggression, violence, and criminal behavior (Edwards et al., 2017; Paulhus et al., 2018; Pfattheicher et al., 2019). These findings also suggest that the Dark Tetrad may be especially prevalent among men expressing particular motives, including exciting and contemptuous sex with a variety of sex workers, commodified and business-like sex, and the desire to have power over and to control sex workers (see Figure 1 for predicted relations). Conversely, the traits of the tetrad may not as clearly typify men seeking sex workers for specific acts due to unfulfilled desires from their partners. The prevalence of the Dark Tetrad dimensions is likely even more diminished among men who buy sex for the purpose of companionship, intimacy, and love.

It is also possible that male clients of outdoor sex work (e.g., street prostitution) embody higher levels of the Dark Tetrad dimensions in comparison to men who purchase indoor services (e.g., escort services). This is because outdoor sex work is characterized by elevated risk, danger, illegal substance use, and the exploitation of women who are more often the targets of violence on behalf of clients (Lowman, 2000; Lowman and Atchison, 2006; Sanders, 2008; Milrod and Monto, 2012, 2017). Indeed, many men who seek female sex workers through internet sexual service providers for indoor prostitution report avoiding outdoor sex workers for these reasons (Milrod and Monto, 2017). Male clients of indoor sex work tend to be older and buy sex for the purpose of companionship, love, and intimacy in comparison to men who pay for outdoor prostitution services (Milrod and Monto, 2012, 2017).

It is important to consider evidence that could falsify the predictions delineated in the previous section. The personality trait of honesty-humility shares a negative association with each Dark Tetrad trait (Lee et al., 2013; Meere and Egan, 2017). Therefore, if honesty-humility is positively associated with the motives of exciting and contemptuous sex, commodified sex, or power and control, evidence would run counter to our predictions. Similarly, if men who buy sex in the form of outdoor prostitution express greater honesty-humility than clients who buy sex using indoor services, this would also contradict our proposition.

In future research, it will be important to examine major dimensions of personality, such as the Dark Tetrad traits, among clients, as well as the type of prostitution service they are accessing. Furthermore, many investigators do not assess whether men who have paid for sex have previously been arrested for solicitation of prostitution, which precludes an examination of this potentially confounding variable (Monto and McRee, 2005; Monto and Milrod, 2014). It is also important for researchers to consider the role of random responding when studying variables with values that are not centered around the middle of response scales, such as narcissism and psychopathy (Holtzman and Donnellan, 2017). Failure to take random responding into account for these kinds of variables can lead to inflated and biased effect size estimates, which can contribute to inaccurate inferences about statistical results.

## Conclusion

In the current mini review, we argue that an evolutionary personality perspective can shed unique insight into the personality characteristics of male clients of female sex work. Given that men who display higher levels of socially deviant personality traits (e.g., the Dark Tetrad dimensions) tend to express a penchant for short-term mating, as well as heightened sensation-seeking, impulsivity, sexual risk taking, and criminality, it is likely that many clients of female sex workers possess similar personalities. These relations may be particularly apparent among male clients espousing specific motives (e.g., power and control), as well as those men who seek outdoor prostitution services. Nonetheless, there is a dearth of research on the personality characteristics that typify men who buy sex from those who do not. Empirical work on this topic is important because it can be used to better inform lawmakers, health professionals, and sex workers regarding the kinds of men who purchase sex, as well as the risks and dangers associated with involvement in particular kinds of prostitution.

## Author Contributions

AD took the lead role in determining the focus of the submission, conducting the literature review, and writing the manuscript. TV and SA provided important guidance in helping to writing and editing the manuscript in preparation for submission. All authors contributed to the article and approved the submitted version.

## Conflict of Interest

The authors declare that the research was conducted in the absence of any commercial or financial relationships that could be construed as a potential conflict of interest.
